# How to Determine the Early Warning Threshold Value of Meteorological Factors on Influenza through Big Data Analysis and Machine Learning

**DOI:** 10.1155/2020/8845459

**Published:** 2020-12-02

**Authors:** Hui Ge, Debao Fan, Ming Wan, Lizhu Jin, Xiaofeng Wang, Xuejie Du, Xu Yang

**Affiliations:** ^1^Chinese Center for Disease Control and Prevention, 102206 Beijing, China; ^2^School of Computer Science and Technology, Beijing Institute of Technology, 100081 Beijing, China

## Abstract

Infectious diseases are a major health challenge for the worldwide population. Since their rapid spread can cause great distress to the real world, in addition to taking appropriate measures to curb the spread of infectious diseases in the event of an outbreak, proper prediction and early warning before the outbreak of the threat of infectious diseases can provide an important basis for early and reasonable response by the government health sector, reduce morbidity and mortality, and greatly reduce national losses. However, if only traditional medical data is involved, it may be too late or too difficult to implement prediction and early warning of an infectious outbreak. Recently, medical big data has become a research hotspot and has played an increasingly important role in public health, precision medicine, and disease prediction. In this paper, we focus on exploring a prediction and early warning method for influenza with the help of medical big data. It is well known that meteorological conditions have an influence on influenza outbreaks. So, we try to find a way to determine the early warning threshold value of influenza outbreaks through big data analysis concerning meteorological factors. Results show that, based on analysis of meteorological conditions combined with influenza outbreak history data, the early warning threshold of influenza outbreaks could be established with reasonable high accuracy.

## 1. Introduction

Infectious diseases are a major health challenge for the worldwide population. Since their rapid spread can cause great distress to the real world, in addition to taking appropriate measures to curb the spread of infectious diseases in the event of an outbreak, proper prediction and early warning before the outbreak of the threat of infectious diseases can provide an important basis for early and reasonable response by the government health sector, reduce morbidity and mortality, and greatly reduce national losses. However, if only traditional medical data is involved, it may be too late or too difficult to implement prediction and early warning of an infectious outbreak.

Influenza, commonly known as *the flu*, is an acute respiratory illness caused by influenza viruses A and B, which is a typical infectious disease [1]. It occurs all over the world and causes considerable morbidity and mortality each year. With high transmission speed, frequent pathogen variation, and a wide range of influence, rapid response and prevention of influenza remain a serious global challenge [2, 3]. WHO estimates that influenza affects 5% to 10% of adults and more than 20% of children worldwide each year [4]. About 250,000 to 500,000 people are killed each year by influenza. If we could find a way to scientifically monitor, predict, and provide early warning of influenza, governments can be prepared to prevent the outbreak and spread of influenza as early as possible. Thus, influenza early warning has received great attention from relevant departments.

Recently, medical big data has become a research hotspot and has played an increasingly important role in public health, precision medicine, and disease prediction [5–8]. In this paper, we focus on exploring a prediction and early warning method for influenza with the help of medical big data.

It is well known that influenza disease and transmission are closely related to seasons, regions, weather and environment, demographic factors, and human behavior, among which meteorological factors are the key factors affecting the onset of influenza in a certain area. Integrating meteorological data and influenza incidence data in a certain area through machine learning and data analysis to mine the influence and effect of meteorological factors on influenza incidence is the main focus of this paper.

Traditional epidemiological surveillance systems are likely to have delayed reporting of confirmed cases. Therefore, in this paper, we will study the relationship between the number of influenza cases in the current period and the meteorological factors before a certain period of time, so as to consider the influence of lag and delay of the epidemic monitoring system, thus exploring a more accurate correlation between meteorological factors and influenza outbreaks.

Previous research mainly used machine learning or deep learning algorithms, through correlation analysis and feature selection work; first screened out important predictors such as temperature, rainfall, and relative humidity; and then made a prediction of the number of influenza incidence, but less research has been done on the establishment of influenza early warning thresholds based on meteorological factors [9, 10].

This paper studies how to determine the early warning threshold value of meteorological factors on influenza, thus providing a way to establish prediction and early warning of an influenza outbreak.

Our contributions are as follows:

1. Establishing a preprocessing process to integrate meteorological data and influenza incidence data
2. Selecting important meteorological indicator features for prediction and early warning of influenza outbreaks through correlation analysis and feature construction
3. Building a prediction and early warning method for influenza outbreaks using machine learning and constructing an early warning threshold of meteorological data for influenza outbreaks through data visualization

The following is organized as follows. Related works are presented in Section 2. Our method is discussed in detail in Section 3. Experiments and results are presented in Section 4. The conclusion is given in Section 5.

## 2. Related Works

With the continuous development of the medical and health industry and the strengthening of the importance of public health, more and more attention has been paid to the monitoring, prediction, and early warning of infectious diseases such as influenza in the world, and the methods and technologies used have been continuously improved.

Choi and Thacker used the ARIMA model (Autoregressive Integrated Moving Average Model) in 1981 to estimate pneumonia and influenza mortality, one of the earliest studies on time series [11].

The percentage of deaths associated with pneumonia and influenza was used as an evaluation index to quantify the impact of influenza on mortality. The experimental results showed that the ARIMA model was more specific than the rule based on the regression model. The model can predict the expected mortality of pneumonia and influenza more accurately, but the factors considered in this study are far too less.

The study of Ugarte et al. [9] in 2010 and the study of Paul and Held [10] in 2011 all adopted the method of applying statistical methods to multivariate time series of infectious disease counts. The latter also introduces specific regions and possibly space-related random effects to explain different levels of incidence or changes in the spread of pathogens across regions.

Conesa et al. used a Bayesian hierarchical Poisson model with hidden Markov structures in 2015 to detect influenza epidemics [12]. By automatically monitoring influenza-related data, they detect epidemics immediately at the outbreak and predict trends in influenza epidemics and outbreaks to generate sensitive, specific, and timely warning alerts.

Marquez and Barron have created an intelligent system to support the diagnosis of influenza using the relevant factors based on historical data of the Mexican population [13]. They proposed to support the first clinical diagnosis with machine learning methods.

Some researchers have also adopted more novel techniques or included other influencing factors to analyze such problems.

Since there are many uncertain factors affecting avian influenza outbreaks, [14] has used the classification model (OOC) to solve the task of avian influenza outbreak prediction.

Dai et al. [15] presented an unsupervised word embedding-based clustering method. They try to use Twitter data to perform surveillance of influenza.

[16] combines CDC statistics, Google Trends web search data, and King Net national medical diagnosis and consultation records to propose a linear prediction framework that demonstrates that a large amount of online social behavior information can be used to indirectly monitor influenza activity.

However, due to the limitations of the linear model itself, the prediction effect is relatively general. There are also many studies on the effect of meteorological factors on influenza-like cases.

[17] compared the model error and sample fitting accuracy of the common regression model and backpropagation neural network based on the genetic algorithm and modeled the high and low flu seasons, respectively.

[18] used artificial neural networks to predict seasonal influenza epidemics in Tehran. The dataset used contains climatic characteristics such as temperature, humidity, precipitation, wind speed, sea level pressure, and the number of patients (total number of referrals and number of patients with flu-like diseases). Different loss functions are defined. The results show that the model provides a satisfactory prediction possibility.

Venna et al. proposed to use long short-term memory- (LSTM-) based multistage forecasting for influenza forecasting [16]. They try to use the LSTM method to capture the temporal dynamics of seasonal flu. And they proposed a technique to capture the influence of external variables that include geographical proximity and climatic variables such as humidity, temperature, precipitation, and sun exposure.

Based on the theory of the Generalized Additive Model (GAM) and the mathematical model based on nonlinear regression, the influence of meteorological factors on the change of influenza-like cases in Urumqi is analyzed in [17]. The results of the single-factor model showed that the difference of all influencing factors was statistically significant, and the monthly sunshine hours, monthly average relative humidity, and monthly average temperature were the risk factors that caused the change of influenza-like cases. The results of the multifactor model show that only the monthly mean relative humidity and the monthly mean temperature are statistically significant.

Jhuo et al. [18] have used the meteorological and pollution parameters and acute upper respiratory infection (AVRI) outpatient number as input to a multilayer perceptron (MLP) to predict the patient number of influenza and the associated pneumonia in the following week. The meteorological parameters they used are temperature and relative humidity, and air pollution parameters are Particulate Matter 2.5 (PM 2.5) and Carbon Monoxide (CO).

We have summarized all those works in Table 1.

## 3. Method

### 3.1. Overview

In this work, we combine the influenza incidence data and meteorological data of a province in China in the past four years, to explore an effective early warning method based on machine learning and big data algorithms, thus providing useful information for influenza prevention in other regions of China.

The whole framework consists of three main parts:

1. *Data Preprocessing*. Including the collection of meteorological data from the internet; cleaning and integrating influenza incidence data and meteorological data; normalization and exploratory analysis of data; data tagging.
2. *Correlation Analysis and Feature Selection*. More complex features are constructed according to domain knowledge, the importance of feature calculation is calculated by the single-factor analysis method, and the feature selection is carried out by the Filter and Embedding combination algorithm.
3. *Model Construction*. Feed data into the decision tree model, adjust the model parameters, construct the prediction model, and optimize the prediction model.

### 3.2. Data Preprocessing

#### 3.2.1. Data Collection and Data Cleaning

The meteorological data is collected from the internet. This work uses Python Requests library and crawler framework Scrapy to collect meteorological data from the National Greenhouse Data System. When crawling meteorological data, first determine the crawling area, then use the *urlencode* function to send a HTTP request to get the corresponding *station*_*id* of the weather station in this area, and then use this *id* as the parameter of the *getWeatherData* request, plus the required date, to send a HTTP request.

Features of collected meteorological data are shown in Table 2:

#### 3.2.2. Data Tagging

In order to train the data models, we need to annotate the original data. The data of influenza incidence and the local meteorological data collected were integrated before tagged.

There are two basic ideas for studying the early warning threshold of meteorological factors. One is to take the daily number of influenza incidence as the explanatory variable, that is, dependent variable, to treat and solve the problem as a regression problem in machine learning, to train the model and to predict the number of future influenza incidence, and to issue an early warning when the number of predicted cases is greater than a certain threshold.

The second is to transform the continuous number of influenza cases into discrete labels of 0 and 1 by means of specific data tagging methods. The data tagging method used here is to define the threshold of influenza outbreaks and to measure whether the current incidence represents an influenza outbreak. After the tagging is completed, the problem can be solved as a classification problem in machine learning, while when the data is predicted, the dates predicted as 1 are regarded as the dates that need to be issued an early warning.

Because influenza has typical seasonal characteristics, it is not so reasonable to compare the predicted continuous values with a specific threshold according to the first idea. And if we divide the data by season and train multiple models, it complicates the problem. Compared with the first idea, the second one is more understandable and easier to implement. After comprehensive consideration and comparison, it is decided to choose the second one as the way to solve the problem in this paper.

According to the specific problem of influenza outbreaks, this paper proposes three methods of data tagging:

- *Moving Percentile Method*. The moving percentile method compares the number of cases in the local current observation cycle with its corresponding historical baseline data in real time. If the number of cases occurring during the current observation cycle reaches or exceeds the warning threshold, an influenza outbreak is considered; that is, the data label is defined as 1. For example, if the number of years of retrospective history is 3 years, the calculation period is 7 days, moving by day, and the historical period rocking back and forth is two reference periods. Suppose we set an early warning threshold for influenza outbreaks to P80; set the label to 1 only if the number of cases within the current observation period (7 days) is greater than or equal to 80% of the historical baseline data; otherwise, set to 0.
- *Monthly Upquartile Marking*. The monthly upquartile marking, by definition, defines the label of the data corresponding to those dates in which the number of cases per month exceeds the monthly upquartile as 1.
- *Dual Cycle Daily Marking*. Through the exploratory analysis of influenza incidence data, it can be found that one year can be divided into two different cycles according to the number of cases per month. The first cycle is from November to April, which is the most frequent period of influenza; the second cycle is from May to October, which is the low stage of influenza, with an average of about 1/3 of the first cycle. Because of the large gap of influenza incidence data in two cycles, it is a reasonable way to define different data tagging methods for different cycles. The specific definitions are as follows: in the first cycle, there are more influenza cases, with days as the basic unit, and the number of cases per day greater than the upper four quartiles of this cycle is marked as 1; that is, an early warning is required; in the second cycle, the number of influenza cases is less, with days as the basic unit. When the number of cases per day is greater than the 90th percentile of the cycle, mark the data as 1.

### 3.3. Correlation Analysis and Feature Selection

As shown in Table 2, we have collected 10 basic features of meteorological data. In order to achieve the goal of this study, we need to use the feature construction method to process the collected basic meteorological data features to construct more complex data features, in order to explore the relationship between meteorological data and influenza outbreaks from a more comprehensive perspective. Based on the obtained basic meteorological data, we constructed 48 new meteorological data features, mainly considering the delayed effect of meteorological factors on the onset time of influenza.

The purpose of feature selection is to select relevant features that are beneficial for learning algorithms from all features while sifting out irrelevant and redundant features to prevent dimensional disaster problems. Moreover, feature selection can also reduce the difficulty of learning tasks and improve the efficiency of the model.

This work uses a combination of Filter and Embedding for feature selection. We first use Filter for feature selection, calculate the correlation between each feature and output value, remove the obviously irrelevant features, reduce the feature dimension, and then use Embedding to fuse the process of feature selection with the process of classifier learning to select features in the process of learning.

After the feature selection phase, we have selected 26 constructed features and 10 basic features to train the models.

### 3.4. Model Construction

#### 3.4.1. Basic Model Construction

In this work, we want to build a model that could generate early warning of influenza outbreaks based on a combination of meteorological data and influenza incidence data through machine learning and data visualization.

The decision tree algorithm could be used for classification or regression. When the relationship between independent variables and dependent variables is nonlinear or there is an interaction between variables, the effect of the linear model will be poor, and the nonlinear model should be considered. One of the important characteristics of a decision tree algorithm compared with the SVM and BP neural network is interpretability, because the process of constructing a decision tree is equivalent to forming an if-then rule set. According to the data visualization results of the decision tree model, the threshold of meteorological conditions for influenza warning is obtained. Therefore, in this work, a decision tree is used to build the basic model.

The CART decision tree algorithm uses the Gini coefficient as the evaluation standard and replaces the logarithmic operation with the quadratic operation. The smaller the Gini coefficient, the smaller the impurity representing the characteristics, and the decision tree will preferentially select the characteristics with the smallest Gini coefficient when splitting. Compared with the entropy model-based algorithm, the computational complexity of the CART algorithm is much lower. CART only produces two branches on each node, so a binary tree is formed, and each feature can be reused. And the CART algorithm can be used to deal with continuity variables.

And as discussed before, we treat the work of generating early warning of influenza outbreaks as a classification issue; in this work, we choose the CART classification tree algorithm to build the basic model.

The CART classification tree algorithm uses the Gini coefficient to perform feature selection, as described by the following equation:

(1) $$\begin{array}{c} \text{Gini}\left(p\right)=\overset{K}{\underset{k=1}{\sum }}pk\left(1-pk\right)=1-\overset{K}{\underset{k=1}{\sum }}p^{2}k, \end{array}$$

where *K* is the number of classes in the sample, while *p*_*k*_ is the probability that a sample belongs to the *k*th class.

Since we treat the work of generating early warning of influenza outbreaks as a 0-1 two-classification issue, Equation (1) could be further simplified as follows:

(2) $$\begin{array}{c} \text{Gini}\left(p\right)=2p\left(1-p\right), \end{array}$$

where *p* is the probability that a sample belongs to class 0.

For the given dataset *D*, assume the number of classes in *D* as *K*. Define*C*_*k*_as the number of samples that belongs to class*k*. Then, the Gini coefficient of dataset *D* could be calculated as follows:

(3) $$\begin{array}{c} \text{Gini}\left(p\right)=1-\overset{K}{\underset{k=1}{\sum }}{\left(\frac{\left|C_{k}\right|}{D}\right)}^{2}. \end{array}$$

For dataset *D*, when the CART tree splits according to feature *A*, *D* would be divided into *D*_1_ and *D*_2_. Under this situation, the Gini coefficient of *D* would be as follows:

(4) $$\begin{array}{c} \text{Gini}\left(D,A\right)=\frac{\left|D_{1}\right|}{D}\text{ Gini}\left(D_{1}\right)+\frac{\left|D_{2}\right|}{D}\text{Gini}\left(D_{2}\right). \end{array}$$

The calculation of the Gini coefficient is much simpler than that of entropy, especially for the two-classification problem, and the loss of accuracy is also smaller. Furthermore, the decision tree generated by the CART classification tree algorithm is a binary tree. Compared with the multitree formed by other decision tree algorithms, the efficiency is undoubtedly further improved.

The flow of the CART decision tree algorithm consists of two phases: decision tree generation and decision tree pruning. We have used the Cost Complexity Pruning (CCP) strategy to direct the decision tree pruning phase in our work.

#### 3.4.2. Model Optimization

In order to fully exploit the potential of the CART algorithm, several parameters of CART need to be optimized (as illustrated by Table 3).

The *max*_*depth* specifies the maximum depth of the tree; limiting this parameter can ensure that the scale of the early warning model is not too complex. The *min*_*impurity*_*decrease* represents the minimum impurity of the node splitting (i.e., Gini coefficient). Since the impurity of a node decreases when the node splits, the node stops splitting immediately when the value of the impurity is less than the value of this threshold. These parameters, especially the *max*_*depth* parameter of the tree, are very important to limit the size of the decision tree after splitting and reduce overfitting to improve the generalization performance of the model.

Because there are far fewer days of influenza outbreaks and early warning each year than there are no early warning days, the dataset itself has an uneven sample ratio. The sample of label = 0 occupies the majority, and the sample of label = 1 is only a few. For the machine learning model, the uneven proportion of positive and negative samples will lead to the deviation of the results; that is, the effect of the model cannot reach the best, and the accuracy of prediction is not good. For this purpose, it needs to be adjusted by the parameter *class*_*weight*. Our algorithm would calculate and give the appropriate weight to all samples in a class according to the proportion of each class in the whole sample. The *min*_*weight*_*fraction*_*leaf* parameter also plays an important role, since a different class has a different weight. Because upsampling will introduce a large amount of redundant data, downsampling will lose most of the information, so the most common practice is to assign different weights.

The setting of *max*_*depth*, *min*_*impurity*_*decrease*, and *min*_*weight*_*fraction*_*leaf* for the CART algorithm would be decided through experiments, which would be discussed later.

The ensemble learning method combines several simple models to form a more complex and comprehensive model. CART could be optimized through ensemble learning to enhance the stability; however, after ensemble learning optimization, it is not feasible to use a visualization method to interpret the relationship between the early warning threshold of influenza outbreaks and the certain features of meteorological data and influenza incidence data. So, we propose a method to enhance the prediction accuracy and achieve visualized interpretation of the decision of the early warning threshold of influenza outbreaks simultaneously.

Based on the idea of ensemble learning, we provided an optimized model to generate a more accurate prediction of influenza outbreaks based on meteorological data and influenza incidence data by combining CART, XGBoost, and LightGBM. XGBoost (eXtreme Gradient Boosting) is proposed by Tianqi Chen et al. in 2015, which is an optimization on GBDT. LightGBM is another optimization of GBDT, which mainly considers how to reduce the usage of memory and how to reduce the cost of multimachine communication.

**Figure 1:**
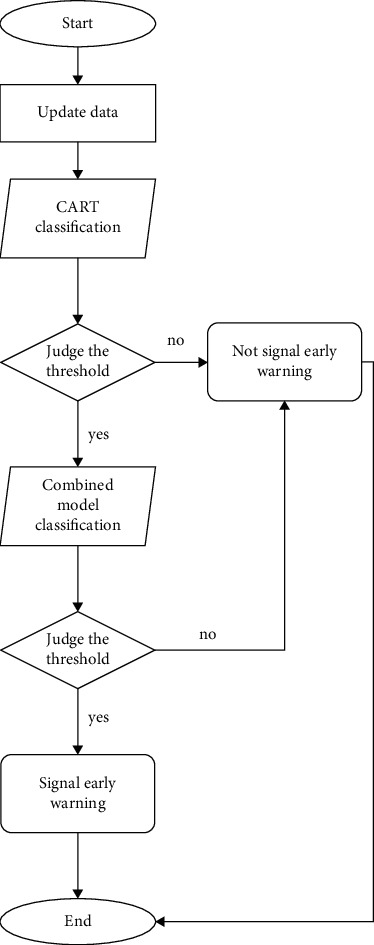
Flow of our method.

The flow of our method is demonstrated in Figure 1.

The CART basic model is used to decide the early warning threshold of influenza outbreaks through data visualization. And if the CART basic model predicts that the early warning threshold is reached according to meteorological data and influenza incidence data, then the combination model formed is used to predict, and if the combination model decides that indeed the early warning threshold is reached, then our model will signal the early warning.

This method reduces the probability of prediction errors in the CART model used alone, but when the meteorological and influenza incidence big data meet the warning conditions of the CART model, they would be sent into the combination model for prediction.

The operation efficiency of the model can be greatly improved. The complexity of this algorithm is comparable with the basic CART algorithm, which is *O*(log*N*), where *N* represents the number of samples in the training set.

## 4. Experimental Results

### 4.1. Experimental Framework

We built our experimental framework using Python 3.5.5. The Hold-Out method divides the dataset *D* into two mutually exclusive subdatasets *D*_1_ and *D*_2_, trains the model on *D*_1_, and tests the effect of the model on *D*_2_. The Hold-Out method is a common method to verify model parameters and evaluate the model effect. Generally speaking, the sample size included in *D*_1_ should account for at least 2/3 of the *D* of the entire dataset. In practice, there is a widely used Hold-Out method [22]: when the data has obvious time series factors, the time of online data is after the offline dataset. In this case, the training set and test set should be divided according to time.

In this work, we comply with the method. Since we have the data for a total of five years from 2012 to 2016, we divided the data from 2012 to 2015 as the training set and the data in 2016 as the test set. Dividing the dataset by year does not destroy the characteristics of the original data, preserves the characteristics of the data distribution to the greatest extent, and avoids the introduction of noise in the segmentation of the data.

The measurement metrics we used in this paper are as follows:

1. *ACC*. Accuracy represents the ratio of the number of samples with the same predicted value as the actual value to the total sample. When the accuracy of the model is higher, it shows that the model prediction results perform better.
2. *f1-score*. f1-score is a more combined metric, which could be calculated as f1‐score = 2 × (precision × recall)/(precision + recall). While recall is calculated as recall = TP/(TP + FN), where TP represents the number of True Positive samples, while FN is the number of False Negative samples. And precision is calculated as precision = TP/(TP + FP), where FP is the number of False Positive samples.
3. *AUC (Area Under Curve)*. AUC is often used to evaluate a two-classification model. AUC reflects a probability value that can intuitively quantify the performance of this classifier. The larger the AUC value, the better the performance of the classifier, and the maximum value is not more than 1. AUC is relatively stable and can better measure the performance of the classifier, that is, the early warning model.

### 4.2. Decision of CART Parameters

Experiments are conducted to decide several most important parameters for CART, as shown in Table 3. The moving percentile method is used to perform data tagging.

#### 4.2.1. Decision of max_depth

Experimental results for different *max*_*depth* are shown in Table 4.

It could be seen from the results that when the *max*_*depth* ≤ 4, the ACC is higher. When the *max*_*depth* > 4, the ACC reduces. f1-score reaches the maximum number when the *max*_*depth* = 4.

And the AUC is also relatively high when the *max*_*depth* = 4. f1-score and AUC reduce as *max*_*depth* becomes larger than 4. We could conclude that if the *max*_*depth* is larger than 4, the model might become overfitting. Thus, we decide that the setting of the *max*_*depth* = 4.

#### 4.2.2. Decision of min_impurity_decrease

Experimental results for different *min*_*impurity*_*decrease* are shown in Table 5.

It could be seen that with the increase of *min*_*impurity*_*decrease*, ACC, f1-score, and AUC show the trend of first increasing and then decreasing. After the value of *min*_*impurity*_*decrease* is greater than 0.08, ACC, f1-score, and AUC all have a large decline. Therefore, it can be judged that the model has the best effect when the parameter is in the range of 0.02 to 0.08. After further evaluation, finally, we set *min*_*impurity*_*decrease* = 0.04.

#### 4.2.3. Decision of min_weight_fraction_leaf

Experimental results for different *min*_*weight*_*fraction*_*leaf* are shown in Table 6.

It could be seen that when the *min*_*weight*_*fraction*_*leaf* = 0.05, f1-score and AUC all reach the maximum value, while ACC is relatively high. Although ACC increases as min_weight_fraction_leaf increases when *min*_*weight*_*fraction*_*leaf* is larger than 0.05, both f1-score and AUC decline largely. Thus, we could conclude that the model gets the best effect when *min*_*weight*_*fraction*_*leaf* is around 0.05. After further evaluation, we set *min*_*weight*_*fraction*_*leaf* = 0.062.

### 4.3. Evaluation of Data Tagging Methods

**Figure 2:**
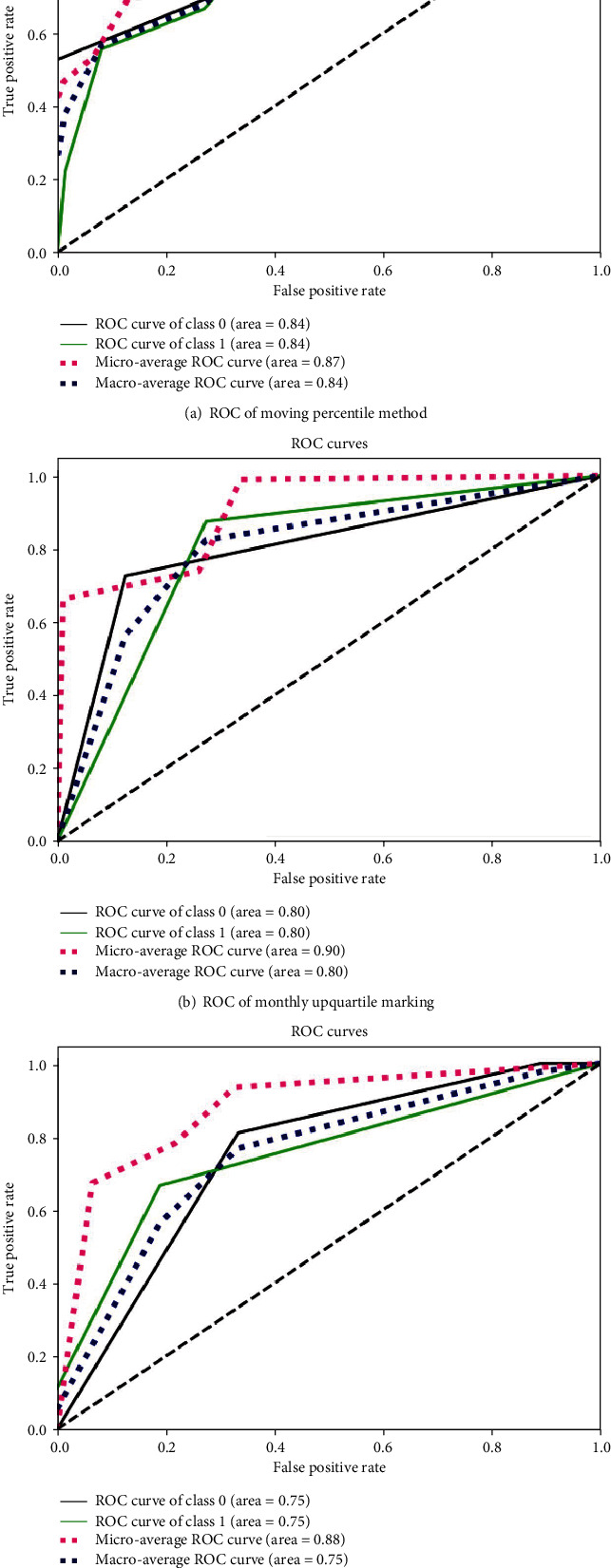
ROC for different data tagging methods.

An experiment is conducted to evaluate the best data tagging method for our model. Results are shown in Table 7 and Figure 2.

Through the comparison, we could decide that the moving percentile method is more suitable for our model.

### 4.4. Definition of the Early Warning Boundary Value of Meteorological Factors on Influenza

**Figure 3:**
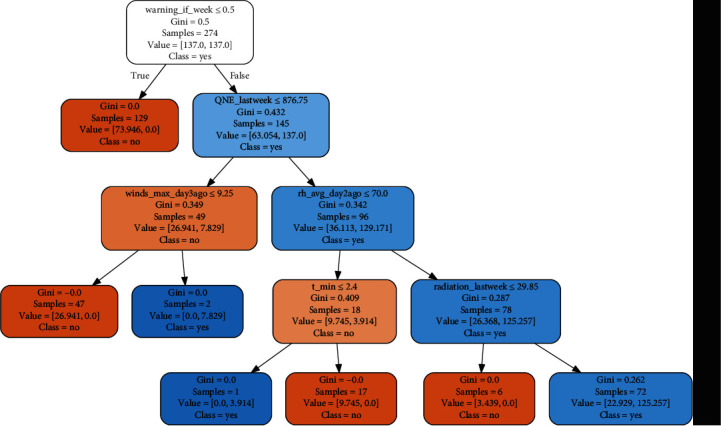
Data visualization of the CART model.

The visualization results of the CART basic model are shown in Figure 3.

As we said before, according to the construction process of the decision tree model, the classification rules can be seen intuitively from the tree structure diagram, and then the meteorological conditions need to be issued when an early warning is given.

Thus, we could generate the early warning boundary value of meteorological factors on influenza based on using the moving percentile tagging method with the CART basic model from Figure 3 as follows: (1) (warning_if_week = 0) and (QNE_lastweek ≤ 876.75 hPa) and (winds_max_day3ago > 9.25 m/s); (2) (warning_if_week = 0) and (QNE_lastweek > 876.75 hPa) and (rh_avg_day2ago ≤ 70%) and (t_min ≤ 2.4°C); and (3) (warning_if_week = 0) and (QNE_lastweek > 876.75 hPa) and (rh_avg_day2ago > 70%) and (radiation_lastweek > 29.85 MJ/m).

It is easy to see that the three conditions are mutually exclusive and only one of them will be satisfied at most. When one condition is satisfied, an early warning is issued.

### 4.5. Evaluation of the Optimized Model

**Figure 4:**
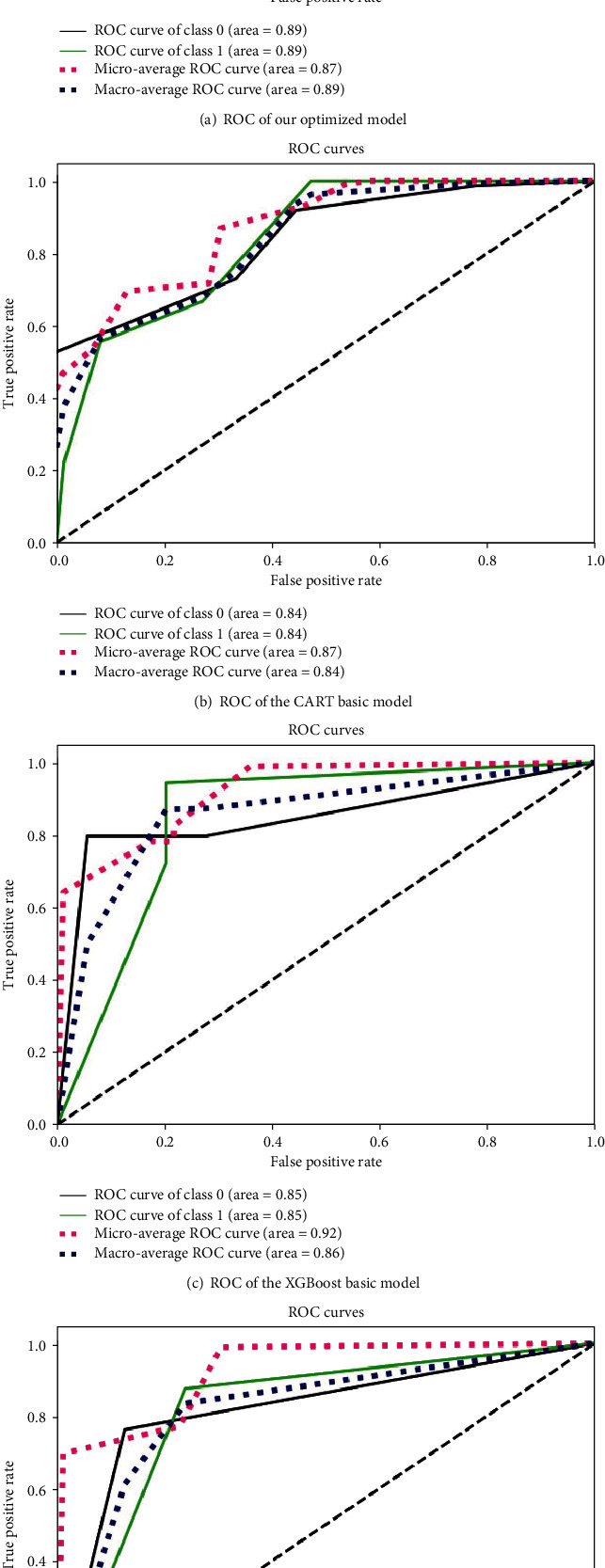
ROC for different basic models.

We use the moving percentile method as the data tagging method. And the comparison between our optimized model and the baseline models is shown in Table 8 and Figure 4.

It could be seen that ACC and AUC of the optimized model are better than those of the CART basic model. But f1-score of the optimized model is smaller than that of the CART basic model. According to our analysis, the mechanism of the optimized model makes the number of samples predicted as 1 become less; thus, the recall rate becomes lower and the f1-score becomes lower. The ACC of CART is relatively low, but the f1-score and AUC are relatively high. The XGBoost model performs well in accuracy and AUC, but the f1-score is relatively low. The LightGBM model is slightly poor in AUC, and the ACC and f1-score tend to be intermediate.

**Figure 5:**
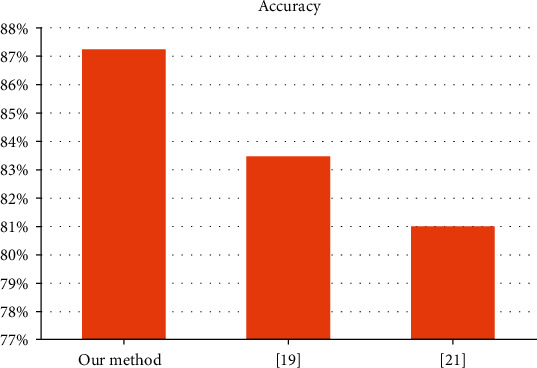
Comparison with other algorithms.

We have also shown a comparison of the accuracy between our method and some state-of-the-art methods in Figure 5.

## 5. Conclusion

In this paper, we try to combine meteorological data and influenza incidence data to build a big data model to determine the early warning boundary value of meteorological factors on influenza. We exploit the data visualization method on the CART basic model to provide a way to generate an early warning threshold for influenza outbreaks based on data analysis of meteorological data. We proposed an optimized model to generate a more accurate early warning signal.

Our approach comes at the expense of slightly reducing the recall rate to improve ACC and AUC and also making full use of the results of the CART model via data visualization. Only when the CART basic model indicates that maybe an early warning should be signaled, then the more complex combination model of XGBoost and LightGBM would be needed. Overall, it is a reasonable scheme according to the evaluation.

Another strategy might be to take the “OR” operation for the construction of the combination optimized model. When at least one model is predicted to be 1, the final prediction result is 1; that is, an early warning is needed. However, the early warning threshold could not be generated through data visualization, thus without interpretability. Also, the computation effort is more. Under realistic conditions, different model combination strategies can be selected according to different needs.

Actually, in this work, we have only introduced key meteorological factors, while the influenza outbreak is also closely related to human flow, intercity migration index, vaccination, emergencies, and other factors. In future work, we would try to establish a more comprehensive way to establish the early warning system for influenza outbreaks.

## Figures and Tables

**Table 1:** Comparison of different influenza-related works.

Reference	Methods	Data	Goal
[11]	ARIMA	Influenza data	Predict trend
[9]	Statistical methods	Influenza data	Predict trend
[10]	Statistical methods	Influenza data	Predict trend
[12]	Bayesian	Influenza data	Predict trend
[13]	Machine learning methods	Influenza data	Support diagnosis
[14]	OOC	Influenza data	Predict outbreak
[15]	Clustering	Social media data	Monitor influenza
[19]	Linear prediction	Medical data and search data	Monitor influenza
[20]	Genetic algorithm	Influenza data	Predict trend
[21]	ANN	Climatic data and influenza data	Predict trend
[16]	LSTM	Geographical data and climatic data	Predict trend
[17]	Nonlinear regression	Meteorological data	Monitor influenza
[18]	MLP	Meteorological data	Predict trend

**Table 2:** Features of meteorological data.

Name	Meaning	Data type	Data unit
t_avg	Daily average temperature	Continuous	°C
t_max	Daily highest temperature	Continuous	°C
t_min	Daily lowest temperature	Continuous	°C
precip	Cumulative precipitation	Continuous	mm
winds_avg	Average wind speed	Continuous	m/s
winds_max	Maximum wind speed	Continuous	m/s
rh_avg	Average relative humidity	Continuous	%
rh_min	Minimum relative humidity	Continuous	%
QNE_hPa	Average air pressure	Continuous	hPa
radiation	Cumulative daily radiation	Continuous	MJ/m^2^

**Table 3:** Critical parameters for CART.

Name	Meaning	Data type	Default value
max_depth	The maximum tree depth	None	
min_impurity_decrease	The minimum impurity for node splitting	0	
min_weight_fraction_leaf	The minimum weight of a leaf node	0	
class_weight	The weight of a class	None	

**Table 4:** Evaluation of max_depth for CART.

max_depth	ACC	f1-score	AUC
2	0.8361	0.6562	0.8019
3	0.8126	0.6793	0.7798
4	0.8135	0.7087	0.7943
5	0.7621	0.6315	0.7109
6	0.7709	0.6107	0.6954
7	0.7891	0.6051	0.6598

**Table 5:** Evaluation of min_impurity_decrease for CART.

min_impurity_decrease	ACC	f1-score	AUC
0	0.8135	0.7087	0.7943
0.005	0.8135	0.7087	0.7943
0.01	0.8143	0.7165	0.8029
0.02	0.8177	0.7254	0.8087
0.05	0.8268	0.7301	0.8109
0.08	0.7521	0.6342	0.7651
0.1	0.7196	0.6072	0.7535

**Table 6:** Evaluation of min_weight_fraction_leaf for CART.

min_weight_fraction_leaf	ACC	f1-score	AUC
0	0.8291	0.7370	0.8153
0.01	0.8043	0.6909	0.7733
0.02	0.8105	0.7144	0.7992
0.05	0.8358	0.7451	0.8208
0.1	0.8470	0.6369	0.7384
0.2	0.8578	0.6882	0.7572
0.3	0.7329	0.6153	0.7023

**Table 7:** Evaluation of different data tagging methods.

Data tagging method	ACC	f1-score	AUC
Moving percentile method	0.8586	0.7610	0.8429
Monthly upquartile marking	0.8317	0.6963	0.7967
Dual cycle daily marking	0.8391	0.7129	0.7508

**Table 8:** Comparison between our model and baseline models.

Method	ACC	f1-score	AUC
Optimized model	0.8721	0.7381	0.8709
CART	0.8586	0.7610	0.8429
XGBoost	0.8804	0.6998	0.8561
LightGBM	0.8735	0.7321	0.8224

## Data Availability

Requests for data (6/12 months after publication of this article) will be considered by the corresponding author.
